# Evaluation of Cross Immunity and Histopathological Findings in Experimentally Infected BALB/c Mice with *Neospora caninum* and *Besnoitia caprae*

**Published:** 2013

**Authors:** M Namavari, A Oryan, F Namazi, M Kargar, M Mansourian, A Rahimian, Y Tahamtan

**Affiliations:** 1 Razi Vaccine and Serum Research Institute, Shiraz, Iran; 2 Department of Pathology, School of Veterinary Medicine, Shiraz University, Shiraz, Iran

**Keywords:** *Neospora caninum*, *Besnoitia caprae*, BALB/c mice, Cross reaction

## Abstract

**Background:**

Caprine besnoitiosis is an economically important disease of goats. *Neospora caninum*, another coccidian parasite of worldwide distribution, infects several animal species and is a major cause of abortion in cattle. Combined infections of *N. caninum* and *Besnoitia caprae* can occur in geographical areas endemic for both species of parasite in goats. This experiment was conducted to investigate the possible cross-immunity between these two infections in experimentally infected BALB/c mice.

**Methods:**

Forty BALB/c mice were divided into four equal groups. The mice of Groups 1 and 4 were inoculated with 1×10^6^ live virulent tachyzoites of *N. caninum* (NC-1), while animals of Groups 2 and 3 were inoculated with sterile tissue culture medium. Each mouse in Groups 1 and 2 was challenged 28 days later with 1×10^6^ live virulent bradyzoites of *B. Caprae* (BC-1).

**Results:**

Following the challenge, the mice in Groups 1 and 2 showed 100% morbidity and 100% mortality within 9 days post infection, while all the animals of Groups 3 and 4 remained alive. The dead animals were necropsied. The survivors (mice in Group 3 and 4) were euthanized 9 days after inoculation and the gross and histopathological lesions in different organs were investigated.

**Conclusion:**

Immunization and challenge experiments with lethal dose of *B. caprae* in the highly susceptible BALB/c mice showed no cross-protection between *N. caninum* and *B. caprae*.

## Introduction

Besnoitiosis and neosporosis are common in a wide range of domestic and wild animals (1–5). They are coccidian parasites belonging to the family Sarcocystidae. Goats could be intermediate host for both parasites. *Besnoitia caprae* is a causative agent which results in high morbidity and low mortality in goats (2, 6). The disease occurs as an epizootic and is associated with skin damages, orchitis, epididymitis and male infertility, granuloma formation in the eyes, neonatal death, and abortion (7–9). Extensive skin damage, condemnation of the organs of the infected carcasses and the adverse effects of the parasite on growth rate and weight gain in association with reduction in male fertility makes this disease of great economic concern to the mutton and leather industries and animal breeders (6, 9–11).

The life cycle of *B. caprae* is not clear and the intermediate host range and the definitive host(s) are not known. There is also a controversy between the investigators in the main natural route(s) of transmission of this protozoon from the definitive host(s) to the intermediates (6, 7). The taxonomy of this genus is also poorly defined, and is based largely on the presence of characteristic bradyzoite-filled cysts in the intermediate host and on the ultrastructural characteristics of the bradyzoites (12, 13). Oryan et al. (14) demonstrated significant differences between the two isolates of *B. caprae* and showed that the inbred BALB/c mice infected with high dose of BC-1 died and those infected with lower doses showed consistent pathological lesions. While all the mice that were infected with different doses of BC-2 remained alive and when were euthanized after 40 DPI, no histopathological changes was seen in the tissue sections of their organs. It is not known whether humans can be infected with *Besnoitia* species, but the wide variety of mammals that have recently been shown to be infected suggests that humans are also at risk.

*Neospora caninum* is another coccidian parasite that has been identified in a wide range of animal species (15) and has been recognized as a significant cause of abortion in cattle in many parts of the world (4, 16–18) and thus it is an economically important disease affecting cattle herds (19, 20). Natural infection with *N. caninum* has been reported in dogs, cattle, goats, sheep, horses and deer (15). It can be the causative agent of abortion or neonatal mortality in goats and several infected cases with *N. caninum* have been described in this animal species (5, 21–24). However, the rates of *N. caninum* infection and the significance of the disease in this species have been poorly investigated. The role of *N. caninum* as a causative agent of natural abortion in small ruminants needs to be investigated, since their experimental inoculation with *N. caninum* during pregnancy causes a condition very similar to that observed in cattle (25). *N. caninum* has a worldwide distribution (26), while *B. caprae* has only been reported from Iran (1, 6) and Kenya (2, 10). Both neosporosis and besnoitiosis caused by *N. caninum* and *B. caprae*, respectively, have been recognized as economically important diseases with considerable impact on the livestock industry.

Similarities between the intermediate and definitive hosts of *N. caninum* and some of the *Besnoitia* species suggest a close relationship between these two organisms. The prevalence rate of caprine besnoitiosis is reported from 19% to 25% in southern and eastern areas of Iran (6, 9, 13). The prevalence rate of neosporosis at different geographical areas of this country is reported from 11.3% to 46.0% in dogs (18, 27) and from 9.9% to 46.0% in dairy cattle (17, 28, 29).

Since infection with either parasite can lead to herd reproduction infertility, although for different reasons, for example, abortion (5, 30–32), or male infertility (8, 9, 33), it is important from the stand point of herd management to know whether there is serological cross-reactivity between these related parasites. Although the changes in the biochemical and hematological parameters in the naturally infected domestic goats by *B. caprae* were studied previously (34, 35) but no report is yet available on the cross immunity between *B. caprae* and *N. caninum*, as another apicomplexan parasite.

Therefore, the present study was undertaken to determine whether infection with *N. caninum* would protect mice against *B. caprae* and thereby to better define the relationship between these two protozoan parasites of mammals and to investigate the possible degree of cross-immunity between these protozoan parasites.

## Materials and Methods

### Isolation of Besnoitia caprae

Isolation and cryopreservation of *B. caprae* were done as described by Oryan et al. (14). The bradyzoites of BC-1 were tested for viability with trypan blue (≥ 95% viable).

### Cultivation of N. caninum tachyzoites

The tachyzoites of *Neospora caninum* (NC-1) were propagated from Razi Vaccine and Serum Research Institute, Tehran, Iran. The tachyzoites used for inoculations were grown in and harvested from Vero cell monolayers in 25 cm^2^ canted neck tissue culture flasks. The monolayers were then disrupted using a sterile cell scraper; the parasites were then counted in a Neubauer haemocytometer and resuspended in PBS to produce inoculate containing 1×10^6^ tachyzoites/0.5 ml.

### Experimental infection of laboratory animals

Forty female BALB/c mice, approximately 10 weeks old, were randomly divided into four groups of 10 animals each and similarly were maintained on standard mouse diet with no limitation of access to food or water.

Each mouse in the first and Fourth groups received 1×10^6^ live virulent tachyzoites of *N. caninum*, and each animal of the Groups 2 and 3 was inoculated with sterile tissue culture medium. Twenty eight days later, each mouse of the Groups 1 and 2 was intraperitoneally inoculated with 1×10^6^ live virulent bradyzoites of *B. Caprae* (BC-1) and the animals of Group 3 were kept as uninfected controls.

### Gross and histopathological examination

When a mouse died it was quickly necropsied and different organs were carefully examined. Tissue samples from the brain, lungs, heart, liver, spleen, limb's muscles and skin were collected for histopathological studies. The animals of Groups 3 and 4 were euthanized on day nine post challenge of group 1 and 2 with bradyzoites of BC-1. The samples were fixed in 10% neutral buffered formalin, embedded in paraffin and sections of 5 µm in thickness were stained with haematoxylin and eosin and studied by an ordinary light microscope (Olympus, Tokyo, Japan).

## Results

None of the mice inoculated with the live virulent tachyzoites of *N. caninum* isolate (animals of Group 1) developed clinical signs of disease before inoculation with BC-1while infection with bradyzoites of BC-1resulted in a fatal infection. All the animals of the Groups 2, 3 and 4 remained alive and showed no clinical symptom till 28 days after inoculation of culture medium. Following challenge of the BC-1bradyzoites, the mice of Group 1 and 2 showed 100% morbidity and 100% mortality within 9 days post-challenge. However, all the animals of Groups 3 and 4 were alive and healthy up to end of the experiment.

**Fig. 1:**
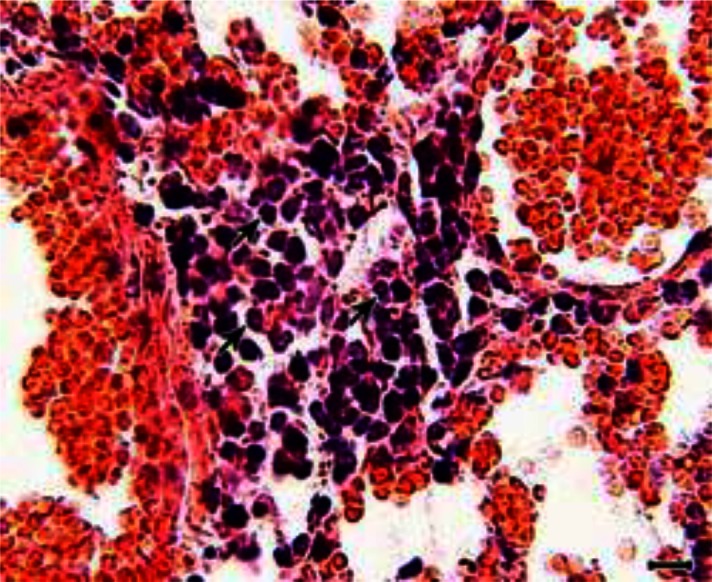
A histopatholgy section from the lungs of an animal of Group 1. Hyperemia, hemorrhages and interstitial pneumonia with mononuclear cell infiltration (arrows) are seen in the lung section (H and E, scale bar = 20 µm)

Macroscopically, pneumonia were present in the mice of Groups 1 and 2 while all the organs of the mice in group 4 and control group (Group 3) were grossly normal. At histopathological level, hemorrhages, hyperemia, and mild interstitial pneumonia with mononuclear cell infiltration in the wall of the alveoli with peribronchitis and peribronchiolitis was present in the lungs of the animals of Groups 1 and 2 (Fig. 1).

**Fig. 2:**
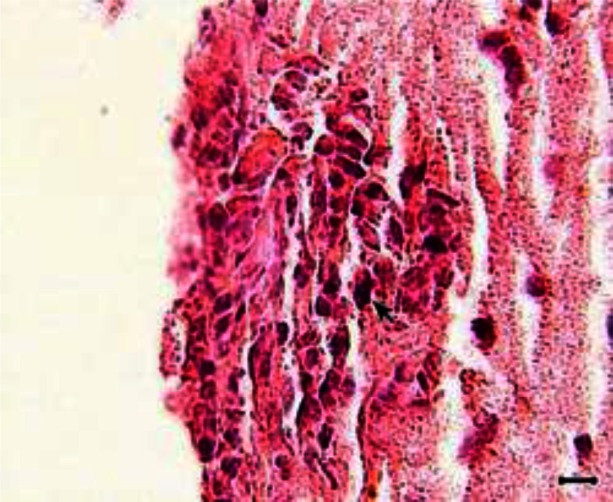
Brain section from a mouse of Group 2. Mild gliosis is evident (arrow) (H and E, scale bar = 20 µm)

**Fig. 3:**
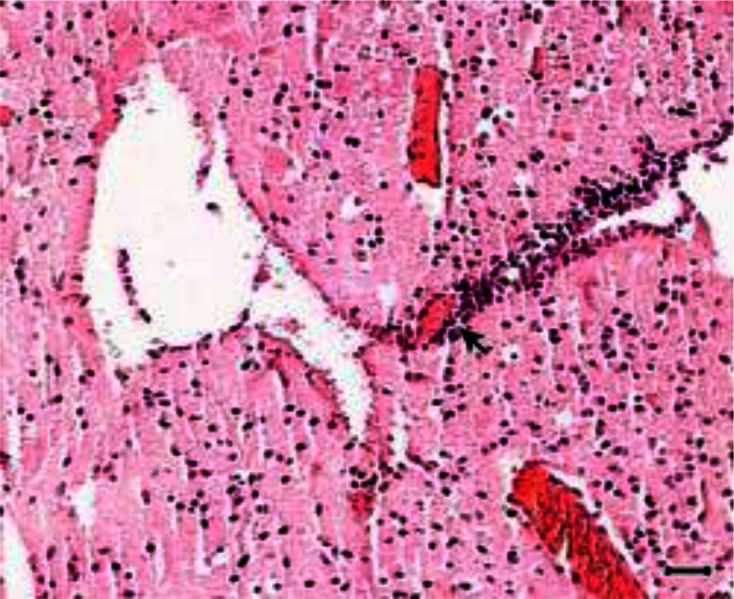
Perivascular cuffing (arrow) with hyperemia is seen in brain section of a mouse of Group 2 (H and E, scale bar = 85 µm)

**Fig. 4:**
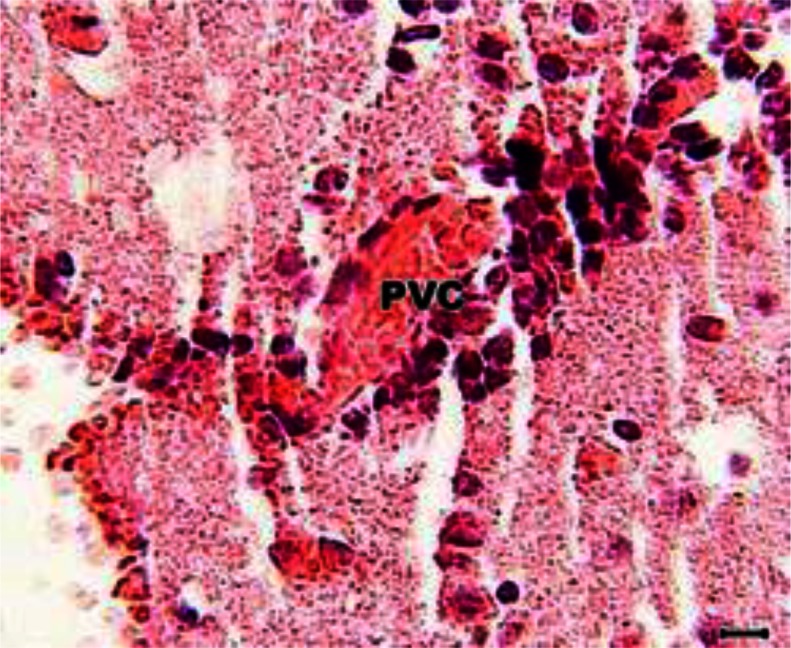
Higher magnification of Fig. 3. PVC (perivascular cuffing) (H and E, scale bar = 20 µm)

The peritoneal cavity of the animals of Groups 1 and 2 showed chronic peritonitis. The brain of the animals of both groups also showed mild gliosis (Fig. 2); however, perivascular cuffing was evident only in animals of Group 1 (Figs. 3 and 4).

The mice inoculated only with *N. caninum* (group 4) represented mild perivascular cuffing in brain sections. No macroscopic or microscopic lesions were evident in different organs of the control animals. No cysts or tachyzoites was evident in the tissue sections of liver, lungs, brain, skin, eyes, gastrointestinal tract, or other examined organs of the animals.

## Discussion

This study demonstrated that mice infected with *N. caninum* were not protected against a lethal challenge with BC-1****. Absence of cross-reactivity between anti *N. caninum* sera and the *B. caprae* specific surface antigen may be interpreted to further support the contention that *N. caninum* has different surface antigens altogether and is not suitable for immunizing domestic animals against besnoitiosis. This finding is in accordance with those of Shkap et al. (36) for another species of *Besnoitia*. They showed that the serological cross-reaction between *N. caninum* antibody and *B. besnoiti* antigen was not considered significant for field diagnosis because it occurred below the serum dilution cutoffs that actually happen in nature and it is stated to be 1:200 for *N. caninum* (15) and 1:256 for *B. besnoiti*. Mouse is a relevant and suitable animal model for maintenance of *N. caninum* infection and has been commonly used as a model in experiments conducted to study the biology of this parasite (37, 38). Similar to the present findings the interpretation of the minor serological cross-reactivity between *N. caninum* and *B. besnoiti* was not supported by the challenge experiments performed in mongolian gerbils (36). However, the mongolian gerbils are highly susceptible to oral infection with the oocysts of *N. caninum* (3, 39). In addition, it was previously showed that Gerbils are highly susceptible to intraperitoneal infection with *B. besnoiti* too (40).

Similar to the present findings, no significant cross-reactivity of *N. caninum* with some of other taxonomically related coccidian parasites has been reported using fluorescent antibody technique and ELISA (41, 42). However, cross reaction between some other species of apicomplexa has previously been reported. Frenkel and Dubey (43) showed that after a challenge with *Toxoplasma gondi i*oocysts this parasite was not lethal for the hamsters that were previously immunized with H*ammondia hammondi* and they became immunized and survived. Presence of viable *Toxoplasma* cysts in the brains of mice and hamsters immunized with *H. hammondi* and challenged with *T. gondii* indicated that *Toxoplasma* was able to multiply in *H. hammondi*-infected animals but at a slower rate than in *H. hammondi*-free mice. Emanuel and Dubey (44) stated that the number of *Toxoplasma* cysts in the brain of the mice or hamsters immunized with *H. hammondi* and in unimmunized animals may be used as another indicator measure of protection afforded by *Hammondia* against *Toxoplasma*.

Presence of pathologic lesions in the lungs, brain and peritoneum shows that these organs are the targets of *B. caprae* and/or *N. caninum*; however, due to quick death of the animals after challenge with *B. caprae*, the time was not enough for development of the *Neospora* and/or *Besnoitia* cysts. Furthermore, these results agree with the findings of previous *N. caninum* and *B. caprae* mouse model experiments (14, 45, 46), in which showed histopathological lesions in the lungs and brain. Also, interstitial pneumonia with hemorrhages has been observed in the tissue sections of chickens infected with *B. caprae* (47). The mice of Group 4 were alive up to the end of this experiment. Collantes-Fernández et al. (48) also showed none of the mice infected with NC-1 died spontaneously till 63 DPI.

Previous studies showed that the *B. caprae* cysts in goats started to develop from 32 to 40 days after inoculation of the bradyzoites (49, 50). Lack of lesions in skin and eyes shows that, unlike goats, the *Besnoitia* cysts possibly do not localize in dermis, subcutaneous tissues and sclera of the mice. It is also reported that *Neospora* cysts did not develop earlier than 70 days post infection in CBA/Ca mice (51). In another experiment, O'Handley et al. (52) experimentally infected sheep with *N. caninum* and found that all the infected sheep were PCR positive by day 32 post infection and remained positive until the end of the study at 49 day post infection; all the infected sheep contained detectable *N. caninum* DNA in the brain tissue at day 49 post infection. However, unlike with PCR, no pathological lesion or parasite was detected in brain, liver, spleen, heart or other examined organs by routine histopathology and immunohistochemical methods.

## Conclusion

Infection of mice with the present dose of *N. caninum*, does not protect against challenge with lethal doses of *B. caprae*. Morover a possible infection of goat with *N. caninum* should not interfere with serodiagnosis of *B. caprae*. However, further investigation with different dilutions and various animal models should be performed in order to determine the protective effects of different doses of *N. caninum* against *B. caprae* infection.
